# Down-Regulation of Porcine Heart Diaphorase Reactivity by Trimanganese Hexakis(3,5-Diisopropylsalicylate), Mn_3_(3,5-DIPS)6, and Down-Regulation of Nitric Oxide Synthase Reactivity by Mn_3_(3,5-DIPS)_6_ and Cu(II)_2_(3,5-DIPS)_4_

**DOI:** 10.1155/MBD.1999.111

**Published:** 1999

**Authors:** Billynda L. Booth, Eva Pitters, Bernd Mayer, John R. J. Sorenson

**Affiliations:** 1 Department of Medicinal Chemistry College of Pharmacy University of Arkansas for Medical Sciences Campus Little Rock AR 72205 USA; 2 Institut für Pharmakologie und Toxikologie KarI-Franzens-Universität Graz Graz A-8010 Austria

## Abstract

Purposes of this work were to examine the plausible down-regulation of porcine heart diaphorase (PHD) enzyme reactivity and nitric oxide synthase (NOS) enzyme reactivity by trimanganese hexakis(3,5-diisopropylsalicylate), [Mn_3_(3,5-DIPS)_6_] as well as dicopper tetrakis(3,5- diisopropylsalicylate, [Cu(II)_2_(3,5-DIPS)_4_] as a mechanistic accounting for their pharmacological activities.

Porcine heart disease was found to oxidize 114 μM reduced nicotinamide-adenine- dinucleotide-′^3^-phosphate (NADPH) with a corresponding reduction of an equivalent concentration of 2,6-dichlorophenolindophenol (DCPIP). As reported for Cu(II)_2_ (3,5-DIPS)_4_, addition of Mn_3_(3,5-DIPS)_6_ to this reaction mixture decreased the reduction of DCPIP without significantly affecting the oxidation of NADPH.  The concentration of Mn_3_(3,5-DIPS)_6_ that produced a 50% decrease in DCPIP reduction (IC_50_) was found to be 5μM. Mechanistically, this inhibition of DCPIP reduction with ongoing NADPH oxidation by PHD was found to be due to the ability of Mn_3_(3,5-DIPS)_6_ to serve as a catalytic electron acceptor for reduced PHD as had been reported for Cu(II)_2_(3,5-DIPS)_4_. This catalytic decrease in reduction of DCPIP by Mn_3_(3,5-DIPS)_6_ was enhanced by the presence of a large concentration of DCPIP and decreased by the presence of a large concentration of NADPH, consistent with what had been observed for the activity of Cu(II)_2_(3,5-DIPS)_4_

Oxidation of NADPH by PHD in the presence of Mn_3_(3,5-DIPS)_6_ and the absence of DCPIP was linearly related to the concentration of added Mn_3_(3,5-DIPS)_6_ through the concentration range of 2.4 μM to 38μM with a 50% recovery of NADPH oxidation by PHD at a concentration of 6 μM Mn_3_(3,5-DIPS)_6_

Conversion of [^3^H] L-Arginine to [^3^H] L-Citrulline by purified rat brain nitric oxide synthase (NOS) was decreased in a concentrated related fashion with the addition of Mn_3_(3,5-DIPS)_6_ as well as Cu(II)_2_(3,5-DIPS)_4_ which is an extention of results reported earlier for Cu(II)_2_(3,5-DIPS)_4_. The concentration of these two compounds required to produce a 50% decrease in L-Citrulline synthesis by NOS, which may be due to down-regulation of NOS, were 0.1 mM and 8μM respectively, consistent with the relative potencies of these two complexes in preventing the reduction of Cytochrome c by NOS.

It is concluded that Mn_3_(3,5-DIPS)_6_, as has been reported for Cu(II)_2_ (3,5-DIPS)_4_ , serves as an electron acceptor in down-regulating PHD and both of these complexes down-regulate rat brain NOS reactivity. A decrease in NO synthesis in animal models of seizure and radiation injury may account for the anticonvulsant, radioprotectant, and radiorecovery activities of Mn_3_(3,5-DIPS)_6_ and Cu(II)_2_(3,5-DIPS)_4_.


### DOWN-REGULATION OF PORCINE HEART DIAPHORASE REACTIVITY BY TRIMANGANESE HEXAKIS(3,5-DIISOPROPYL-SALICYLATE), $Mn_3$(3,5-DIPS)$_6$, AND DOWN-REGULATION OF NITRIC OXIDE SYNTHASE REACTIVITY BY $Mn_3$(3,5-DIPS)$_6$ AND $Cu(II)_2$(3,5-DIPS)$_4$

Billynda L. Booth[1], Eva Pitters[2], Bernd Mayer[2], and John R. J. Sorenson*[1]

[1] Department of Medicinal Chemistry, College of Pharmacy, University of Arkansas for Medical Sciences Campus, Little Rock, AR 72205, USA; e-mail: sorensonjohnr@exchange.uams.edu
[2] Institut für Pharmakologie und Toxikologie, Karl-Franzens-Universität Graz, A-8010 Graz, Austria

Abstract

Purposes of this work were to examine the plausible down-regulation of porcine heart diaphorase (PHD) enzyme reactivity and nitric oxide synthase (NOS) enzyme reactivity by trimanganese hexakis(3,5-diisopropylsalicylate), [$Mn_3$(3,5-DIPS)$_6$] as well as dicopper tetrakis(3,5-diisopropylsalicylate, [$Cu(II)_2$(3,5-DIPS)$_4$] as a mechanistic accounting for their pharmacological activities.

Porcine heart disease was found to oxidize 114 μM reduced nicotinamide-adenine-dinucleotide-3'-phosphate (NADPH) with a corresponding reduction of an equivalent concentration of 2,6-dichlorophenolindophenol (DCPIP). As reported for $Cu(II)_2$(3,5-DIPS)$_4$, addition of $Mn_3$(3,5-DIPS)$_6$ to this reaction mixture decreased the reduction of DCPIP without significantly affecting the oxidation of NADPH. The concentration of $Mn_3$(3,5-DIPS)$_6$ that produced a 50% decrease in DCPIP reduction ($IC_{50}$) was found to be 5 μM. Mechanistically, this inhibition of DCPIP reduction with ongoing NADPH oxidation by PHD was found to be due to the ability of $Mn_3$(3,5-DIPS)$_6$ to serve as a catalytic electron acceptor for reduced PHD as had been reported for $Cu(II)_2$(3,5-DIPS)$_4$. This catalytic decrease in reduction of DCPIP by $Mn_3$(3,5-DIPS)$_6$ was enhanced by the presence of a large concentration of DCPIP and decreased by the presence of a large concentration of NADPH, consistent with what had been observed for the activity of $Cu(II)_2$(3,5-DIPS)$_4$.

Oxidation of NADPH by PHD in the presence of $Mn_3$(3,5-DIPS)$_6$ and the absence of DCPIP was linearly related to the concentration of added $Mn_3$(3,5-DIPS)$_6$ through the concentration range of 2.4 μM to 38 μM with a 50% recovery of NADPH oxidation by PHD at a concentration of 6 μM $Mn_3$(3,5-DIPS)$_6$.

Conversion of [$^3$H] L-Arginine to [$^3$H] L-Citrulline by purified rat brain nitric oxide synthase (NOS) was decreased in a concentrated related fashion with the addition of $Mn_3$(3,5-DIPS)$_6$ as well as $Cu(II)_2$(3,5-DIPS)$_4$ which is an extention of results reported earlier for $Cu(II)_2$(3,5-DIPS)$_4$. The concentration of these two compounds required to produce a 50% decrease in L-Citrulline synthesis by NOS, which may be due to down-regulation of NOS, were 0.1 mM and 8μM respectively, consistent with the relative potencies of these two complexes in preventing the reduction of Cytochrome c by NOS.

It is concluded that $Mn_3$(3,5-DIPS)$_6$, as has been reported for $Cu(II)_2$ (3,5-DIPS)$_4$, serves as an electron acceptor in down-regulating PHD and both of these complexes down-regulate rat brain NOS reactivity. A decrease in NO synthesis in animal models of seizure and radiation injury may account for the anticonvulsant, radioprotectant, and radiorecovery activities of $Mn_3$(3,5-DIPS)$_6$ and $Cu(II)_2$(3,5-DIPS)$_4$.

### Introduction

In neuronal cells of the brain [1] and many other cell types of other tissues [2] nitric oxide (.N=O, NO) and L-Citrulline (L-Cit) are produced by constitutive cytosolic reduced nicotinamide-adenine-dinucleotide-3'-phosphate (NADPH)-dependent nitric oxide synthase (NOS) oxidation of L-Arginine (L-Arg) at a reductase-oxygenase coupled domain. Roles of NO have been reviewed by Moncada et al. [2], Moncada [3], Lancaster [4], Meller and Gebhart [5], and Nussler and Billiar [6]. It was pointed out by Moncada et al. [2] that as early as 1977 Miki et al. [7] suggested that the role of NO in normal brain function was activation of cytosolic guanylyl cyclase (GC). This activation by NO is now recognized as a feature of its role as a retrograde neurotransmitter [8] that has most recently been well characterized as a signal transduction mechanism within and between different areas of the brain [9]. Inducible NOS activity is also important in immune-mediated white blood cell responses to inflammation, cancer, and infection and cytokine mediation of many organ inflammatory diseases as well as pain [2,10].

Dicopper(II) tetrakis(3,5-diisopropylsalicylate), [$Cu(II)_2(3,5\text{-}DIPS)_4$], has been shown to have antiinflammatory, antiulcer,, anticonvulsant, anticancer, anticarcinogenic, antimutagenic, antidiabetic, analgesic, radioprotectant, and radiorecovery activities and decreases ischemia-reperfusion injury [11 and references therein]. Following the report that $Cu(II)_2(3,5\text{-}DIPS)_4$ inhibits NADPH-dependent mixed function oxidase systems by serving as an electron acceptor, it was studied in the NADPH-dependent porcine heart diaphorase (PHD) system and in a NADPH-dependent tissue staining procedure used to detect NOS activity [10]. These studies revealed that $Cu(II)_2(3,5\text{-}DIPS)_4$ down-regulated PHD wherein PHD continued to oxidize NADPH, without reducing an obligate electron acceptor, dichlorophenolindophenol (DCPIP). It was also found that reducing equivalents derived from NADPH by PHD reduced Cu(II) to Cu(I) in a catalytic fashion causing a decrease in the reduction of DCPIP. These results lead to the provision of indirect evidence that the synthesis of NO by NOS in brain tissue sections was also decreased by incubating these tissue sections in medium containing $Cu(II)_2(3,5\text{-}DIPS)_4$ [10].

Epileptic seizures and radiation injury, both inflammatory disease states, may be associated with excessive production of NO[10]. Like $Cu(II)_2(3,5\text{-}DIPS)_4$, $Mn_3(3,5\text{-}DIPS)_6$ has also been found to be a rapidly acting anticonvulsant agent in preventing Metrazol- and Maximal Electroshock-induced seizures and to have sedative-hypnotic activities with $ED_{50}$ values ranging from 114 to157 μmol/kg of body mass [12]. Like $Cu(II)_2(3,5\text{-}DIPS)_4$, $Mn_3(3,5\text{-}DIPS)_6$ also has radioprotectant and radiorecovery activities in that it also increases survival of whole body gamma-irradiated mice when non-toxic doses of $Mn_3(3,5\text{-}DIPS)_6$ are administered either before or after lethal irradiation [12]. Treatment with 80 μmol $Mn_3(3,5\text{-}DIPS)_6$/kg prior to low dose, $LD_{35}$, irradiation produced 100% survival, a 54% increase in survival, compared to vehicle-treated control mice (65% survival) [12]. Treatment with 40 μmol $Mn_3(3,5\text{-}DIPS)_6$/kg after high-dose, $LD_{72}$, irradiation produced a 229% increase in survival above vehicle-treated control mice (28% survival) [12]. The effective radioprotectant and radiorecovery doses are 1/9 to 1/10 the acutely toxic dose of $Mn_3(3,5\text{-}DIPS)_6$ and thus exhibit a large margin in safety for use as a radioprotectant and radiorecovery agent as found for $Cu(II)_2(3,5\text{-}DIPS)_4$ [13].

Either SOD-mimetic activity, facilitation of *de novo* synthesis of $Cu_2Zn_2SOD$ or MnSOD, or synthesis of other Cu-and Mn-dependent tissue repair enzymes have been suggested as plausible modes of action for $Cu(II)_2(3,5\text{-}DIPS)_4$ and $Mn_3(3,5\text{-}DIPS)_6$ in accounting for their pharmacological activities [13]. It has since occurred to us that $Mn_3(3,5\text{-}DIPS)_6$ might also down-regulate NADPH-dependent enzymes such as PHD and NOS as has been reported for $Cu(II)_2(3,5\text{-}DIPS)_4$ [10].

To examine this question in a system which is less complicated than NOS but shown to be relevant to this enzyme system [10], the reactivity of $Mn_3(3,5\text{-}DIPS)_6$ was examined using PHD. These studies revealed that $Mn_3(3,5\text{-}DIPS)_6$ does down-regulate PHD. This complex as well as $Cu(II)_2(3,5\text{-}DIPS)_4$ were then examined for their ability to decrease the synthesis of L-Cit by authentic NOS using purified rat brain NOS.

We are reporting the down-regulation of PHD by $Mn_3(3,5\text{-}DIPS)_6$ wherein this complex does not inhibit PHD but serves as an electron acceptor in inhibiting reduction of DCPIP, the dye used as the obligate electron acceptor for PHD, and in serving as an electron acceptor this complex may down-regulate rat brain NOS in decreasing the synthesis of L-Cit.

Additional support for the suggestion that $Cu(II)_2(3,5\text{-}DIPS)_4$ may down-regulate NOS is also presented with the demonstration that this complex also decreases the synthesis of L-Citrulline and NO using purified rat brain NOS. It is plausible that the pharmacological activities of $Mn_3(3,5\text{-}DIPS)_6$ and $Cu(II)_2(3,5\text{-}DIPS)_4$ may be due in part to down-regulation of NOS.

### Materials and Methods

Sodium 2,6-Dichlorophenolindophenol monohydrate [DCPIP], Trizma Base, Porcine Heart Diaphorase [9001-18-7] (PHD), and NADPH (all from the Sigma Chemical Co., Milwaukee, WI) and Hoarse Heart Cytochrome C Type III [9007-43-6] and Calmodulin [27-370-7]) (from Sigma, Deisenhofen, Germany) were used as purchased without further purification. Tritiated-L-Arginine

[(2,3,4,5-$^{3}$H)-L-Arg,$^{3}$H-L Arg] and tritiated L-Citrulline [(2,3,4,5-$^{3}$H)-L-Citrulline, $^{3}$H-L-Cit] were obtained from Med Pro (Amersham), Vienna, Austria. Ultraviolet and visible absorbances were measured with a Hewlett Packard 8452A Diode Array Spectrophotometer. All glassware was thoroughly cleaned with either aqua regia or Citronox (Alconox Inc., New York, NY) and all plasticware was metal-free polypropylene. Deionized water, pH 7.5, was used throughout.

A 300 mM solution of Tris buffer and 10 mM solution of DCPIP were prepared in deionized water. A solution of 200 µM $Mn_3$(3,5-DIPS)$_6$ was prepared by dissolving 15.4 mg of $Mn_3$(3,5-DIPS)$_6$($H_2O$)$_{1.25}$ (1531 Da) in 10 mL of ethanol. One mL of this solution was then diluted with 4 mL of ethanol. All concentrations of $Mn_3$(3,5-DIPS)$_6$ are based upon a trinuclear form of this complex, Mn(II)$_3$(3,5-DIPS)$_6$($H_2O$) or Mn(III)$_2$(II)(O)(3,5-DIPS)$_6$ [14-16], since these plausible structural forms of the complex were used to calculate the mass of complex required to prepare these solutions.

For studies employing a low concentration (38 µM) of NADPH (833.4 Da for the tetrosodium salt) and DCPIP (308.1 Da for the monohydrate) the enzyme reaction mixture contained 125 µL of 1.0 mM NADPH and 25 µL of 5 mM DCPIP in 3.125 mL of 300 mM Tris buffer. For studies employing a high concentration (114 µM) of NADPH and DCPIP the enzyme reaction mixture contained 375 µL of 1.0 mM NADPH and 75 µL of 5 mM DCPIP in 2.825 mL of 300 mM Tris buffer. Concentrations of these reactants in these reaction mixtures were arrived at through a series of experiments wherein equimolar concentrations of NADPH and DCPIP gave absorbances that were conveniently measured within the absorbance range of 0 to 2. These reactions were then started with the addition of 25 µL (1U) of PHD.

Visible and ultraviolet spectrophotometric measurements were performed at 600 nm for DCPIP and 340 nm for NADPH with zero absorbance set with buffer. When $Mn_3$(3,5-DIPS)$_6$ was to be added to a reaction mixture, the studied concentration of $Mn_3$(3,5-DIPS)$_6$, or an equal volume of ethanol, was added to buffer to set zero absorbance and eliminate the absorbance due to the 3,5-DIPS ligand, $\lambda_{max}$ = 306 nm.

To determine the effect of $Mn_3$(3,5-DIPS)$_6$ on the PHD enzyme system, a final concentration of 0.0 µM, 3.05 µM, 6.1 µM, or 12.2 µM [200 µL of ethanol or 50 µL, 100 µL, or 200 µL of the 200 µM $Mn_3$(3,5-DIPS)$_6$ solution] was added to the reaction mixture. To examine $Mn_3$(3,5-DIPS)$_6$ as an electron acceptor for NADPH reduced PHD, a concentration of 0.0 µM, 2.4 µM, 4.8 µM, 9.5 µM, 19 µM, or 38 µM $Mn_3$(3,5-DIPS)$_6$ was added to the reaction mixture in the absence of DCPIP. The initial rate of 0.37 nmol/min for NADPH oxidation with the addition of 200 µl of ethanol, used to make the 0 µmol addition of $Mn_3$(3,5-DIPS)$_6$ in the absence of DCPIP, was subtracted from initial rates obtained for additions of alcohol solutions of $Mn_3$(3,5-DIPS)$_6$ used to demonstrate recovery of PHD activity. Initial rates were obtained by measuring changes in absorbance at 5 minutes following the addition of enzyme. All determinations were performed in triplicate and results presented as means $\pm$S.E.M..

Decreases in absorbance at 340 nm for the oxidation of NADPH and 600 nm for the reduction of DCPIP were measured. It was found that the 340 nm absorbance of DCPIP decreased at the same rate as the rate of decrease of the 600 nm absorbance. For the complete NADPH and DCPIP reaction mixture the 340 nm absorbance is due to the combined absorbances of NADPH and DCPIP at 340 nm. The apparent rate of decrease of the 340 nm absorbance for the complete reaction mixture was found to be approximately twice, 0.224 nmol/min, the rate of decrease of the 600 nm absorbance, 0.099, due to the oxidation of NADPH and reduction of DCPIP.

Rat brain NOS was purified from recombinant baculovirus-infected cells as described elsewhere [17]. Synthesis of $^{3}$H-L-Cit and NO from $^{3}$H-L-Arg was determined by incubation of 0.2 to 0.3 µg of enzyme at 37°C for 10 min in 0.1 ml of a 50 mM triethanolamine hydrochloride buffer (pH 7.0) containing 0.1 mM $^{3}$H-L-Arg (~50,000 cpm), 0.2 mM NADPH, 5 µM flavin adenine dinucleotide, 10 µM tetrahydrobiopterin, 0.5 mM $CaCl_2$, and 10 µg calmodulin /ml in the presence of $Mn_3$(3,5-DIPS)$_6$ or Cu(II)$_2$(3,5-DIPS)$_4$ (1048 Da for the dihydrate) added as a dimethylsulfoxide solution, followed by isolation of $^{3}$H-L-Cit by cation exchange chromatography which has been described in detail [18]. All determinations were performed in triplicate except the decrease in L-Cit caused by additions of 3µM, 30µM, or 0.5mM Cu(II)$_2$(3,5-DIPS)$_4$ and 1mM $Mn_3$(3,5-DIPS)$_6$. These were all duplicate determinations except for the addition of 3 µM Cu(II)$_2$ (3,5-DIPS)$_4$ which was a single determination. Except for this single determination data are presented as means $\pm$ S.E.M.

Cytochrome c reductase activity was determined as described elsewhere [19]. Nitric oxide synthase (0.5 μg) was incubated in 0.2 ml of a 50 mM triethanolamine hydrochloride buffer, pH 7.0, containing 0.15 mM NADPH, 3 μM free $Ca^{2+}$, 10 μM $Cu(II)_2(3,5\text{-DIPS})_4$ or 100 μM $Mn_3(3,5\text{-DIPS})_6$ and 0.2 mM cytochrome c at 37° C in the presence of 2 μg of calmodulin. Dimethylsulfoxide solutions of these complexes were used to make additions to the reation mixture. The increase in absorbance at 500 nm was continuously monitored against blank samples containing buffer instead of enzyme, and the amount of enzymatically reduced cytochrome c was calculated using an extinction coefficient of 21 $mM^{-1}$ $cm^{-1}$. Data are results of a single experiment wherein the control was performed in triplicate and duplicate determinations were performed for the addition of both complexes. Results are presented as means ±S.E.M.

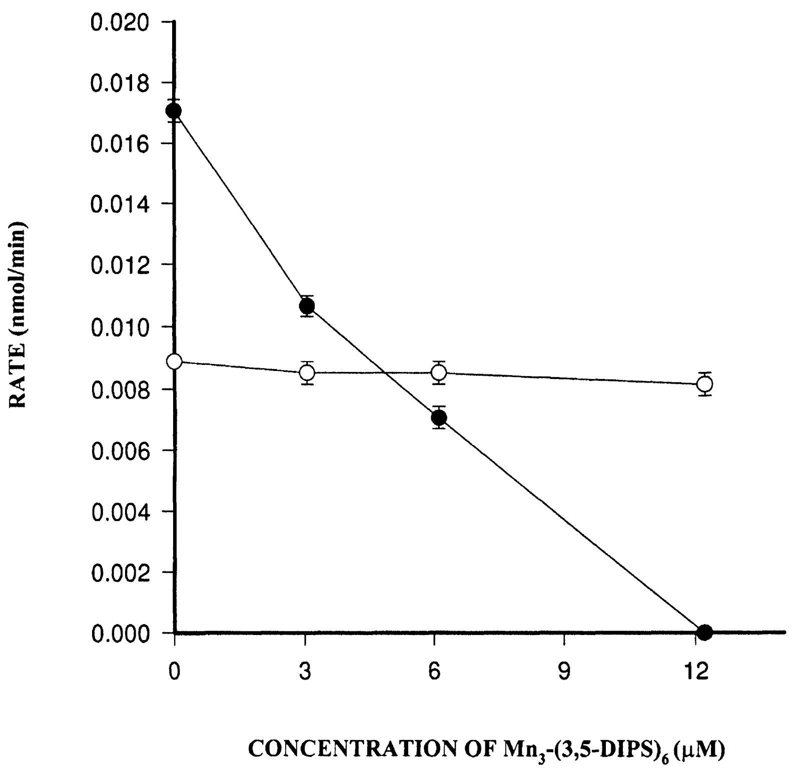

Figure 1. Maintenance of the initial rate of 114 μM NADPH oxidation (O) and the decrease in initial rate of 114 μM DCPIP reduction (●) in the presence of $Mn_3(3,5\text{-DIPS})_6$.

Results

As shown in Figure 1, addition of 1 unit of PHD to a mixture of 114 μM NADPH and 114 μM DCPIP caused the reduction of the obligate electron acceptor DCPIP. The initial rate of decrease in absorbance at 340 nm appeared to be faster than the initial rate of decrease in absorbance at 600 nm due to an absorbance for DCPIP at 340 nm which also decreased at the same rate as the decrease in absorbance at 600 nm with the reduction of DCPIP. Addition of 3 to 12 μM $Mn_3(3,5\text{-DIPS})_6$ produced a concentration related decrease in the initial rate of reduction of DCPIP with little change in the initial rate of oxidation of NADPH. The concentration of $Mn_3(3,5\text{-DIPS})_6$ required to produce a 50% decrease in reduction of DCPIP by NADPH reduced PHD was 5.0 μM. These results demonstrated that PHD oxidation of NADPH occurred in the absence of the reduction of DCPIP, the obligate electron acceptor required for oxidation of NADPH by PHD.

Oxidation of NADPH by PHD in the absence of DCPIP reduction suggested that $Mn_3(3,5\text{-DIPS})_6$ served as the obligate electron acceptor in enabling the continued oxidation of NADPH. This suggestion was examined by determining the oxidation of NADPH in the absence of DCPIP and the presence of increasing concentrations of 2.4 to 38 μM $Mn_3(3,5\text{-DIPS})_6$ to a reaction mixture containing 38 μM NADPH. Recovery of the initial rate of enzymatic oxidation of NADPH was directly related to increasing concentration of added $Mn_3$(3,5-DIPS)$_6$ as shown in Figure 2.

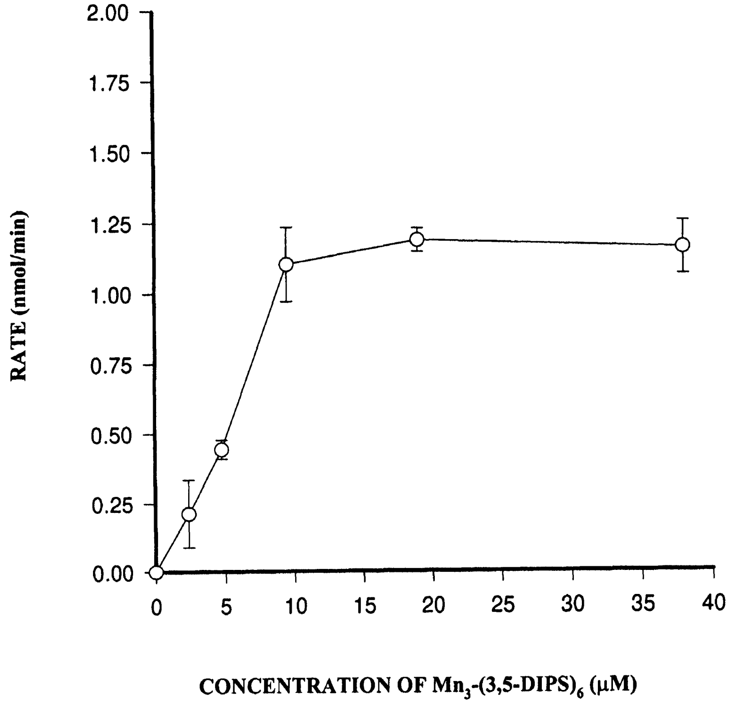

Figure 2. Recovery of the maximum initial rate of oxidation of 38 μM NADPH in the presence of increasing concentration of $Mn_3$(3,5-DIPS)$_6$.

The concentration of $Mn_3$(3,5-DIPS)$_6$ required to produce 50 percent recovery of the maximum initial rate of PHD activity in this system was 6 μM. Under these conditions the role of $Mn_3$(3,5-DIPS)$_6$ appears to be catalytic. This demonstration of enzyme activity recovery is consistent with the suggestion that $Mn_3$(3,5-DIPS)$_6$ does not inhibit PHD in causing the decrease in reduction of DCPIP in the complete NADPH-PHD-DCPIP reaction mixture. The decrease in reduction of DCPIP in the presence of $Mn_3$(3,5-DIPS)$_6$ which enables the oxidation of NADPH supports the suggestion that $Mn_3$(3,5-DIPS)$_6$ does not inhibit PHD but serves to "down-regulate" PHD reduction of DCPIP by serving as an election acceptor for reduced PHD and enabling continued oxidation of NADPH. The decrease in reduction of DCPIP in the presence of $Mn_3$(3,5-DIPS)$_6$ can not be referred to as PHD inhibition and should be viewed as down-regulation of PHD in decreasing the reduction of DCPIP. This interpretation of these results suggested that this complex might also down-regulate NOS.

To examine the possibility that $Mn_3$(3,5-DIPS)$_6$ might down-regulate NOS, purified rat brain NOS was used to measure the conversion of L-Arg to L-Cit in the presence of $Mn_3$(3,5-DIPS)$_6$. As shown in Figure 3, incubation of NOS with concentrations of $Mn_3$(3,5-DIPS)$_6$ ranging from $10^{-8}$M to $10^{-3}$ M caused a concentration related decrease in the conversion of L-Arg to L-Cit with an $IC_{50}$ for the decrease in L-Cit syntheses of 0.1 mM. Additions of $10^{-8}$ M to $10^{-3}$ M Cu(II)$_2$(3,5-DIPS)$_4$ to this enzyme system also produced a concentration related decrease in conversion of L-Arg to L-Cit with an $IC_{50}$ of 8 μM.

These results are consistent with the relative potencies of these two complexes in preventing the reduction of Cytochrome c by NOS as shown in Figure 4. A concentration of 10 μM Cu(II)$_2$(3,5-DIPS)$_4$ was more effective in decreasing Cytochrome c reduction than 67 μM $Mn_3$(3,5-DIPS)$_6$.

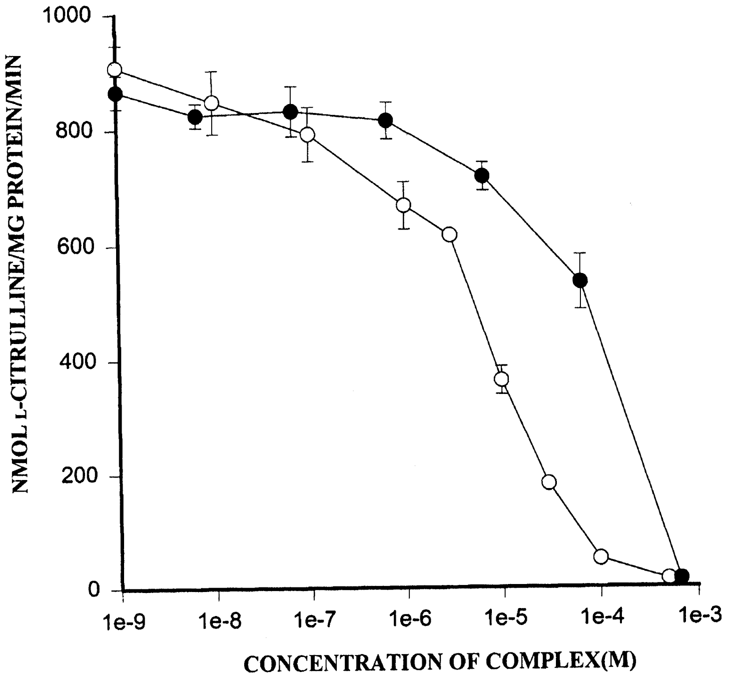

Figure 3. Concentration dependent down-regulation of NOS by $Mn_3(3,5\text{-}DIPS)_6$ (●) and $Cu(II)_2(3,5\text{-}DIPS)_4$ (O).

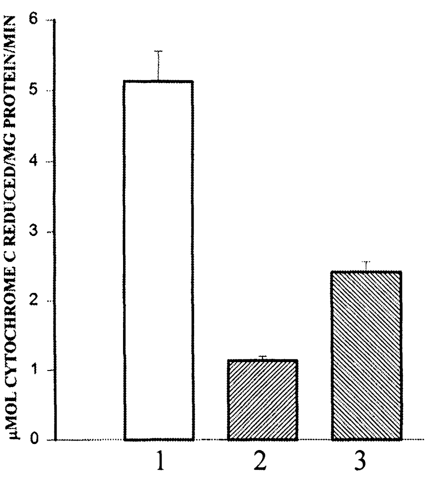

Figure 4. Reduction of Cytochrome c by NOS (1) and the inhibition of Cytochrome c reduction due to the presence of 10 μM $Cu(II)_2(3,5\text{-}DIPS)_4$ (2) or 67 μM $Mn_3(3,5\text{-}DIPS)_6$ (3).

Discussion

Continuous oxidation of NADPH by PHD requires the presence of an electron acceptor. 2,6-dichlorophenolindophenol serves as an electron acceptor in its role as an *in vitro* substrate for the determination of PHD activity. The decrease in reduction of DCPIP in the presence of $Mn_3(3,5\text{-}DIPS)_6$ with continued oxidation of NADPH suggested that $Mn_3(3,5\text{-}DIPS)_6$ served as an electron acceptor in down-regulating, but not inhibiting, the reduction of DCPIP by NADPH-reduced PHD. Evidence for this down-regulation was obtained when the removal of DCPIP and addition of increasing concentration of $Mn_3(3,5\text{-}DIPS)_6$ caused a recovery of NADPH oxidation by PHD, demonstrating that PHD activity was not inhibited by $Mn_3(3,5\text{-}DIPS)_6$.

When recovery was determined for the enzyme system containing 114 μM NADPH and 1 unit of PHD, the addition of up to 48.8 μM $Mn_3(3,5\text{-DIPS})_6$ did not yield 100% recovery of the initial rate of NADPH oxidation. However, the system containing 38 μM NADPH and 1 unit of PHD permitted maximum recovery of NADPH oxidation and 50 percent recovery was found for the addition of 6 μM $Mn_3(3,5\text{-DIPS})_6$.

It is suggested that $Mn_3(3,5\text{-DIPS})_6$ functions in a catalytic role as the electron acceptor in recovery of PHD activity. In the proposed mechanism in which $Mn_3(3,5\text{-DIPS})_6$ serves as an electron acceptor in down-regulation of PHD, Mn(III) is reduced to Mn(II) which is in turn oxidized to Mn(III). As shown in Figure 5, oxidation of Mn(II), the product of PHD reduction, to Mn(III) by dissolved oxygen and the disproportionation of the resultant superoxide [12] and/or hydrogen peroxide [16,20] by $Mn_3(3,5\text{-DIPS})_6$, must be relatively fast reactions compared to the slow rate of oxidation of NADPH and reduction of DCPIP by PHD. This rationale for the catalytic role of $Mn_3(3,5\text{-DIPS})_6$ is the same as the proposed role of $Cu(II)_2(3,5\text{-DIPS})_4$ in down-regulating NOS[10]

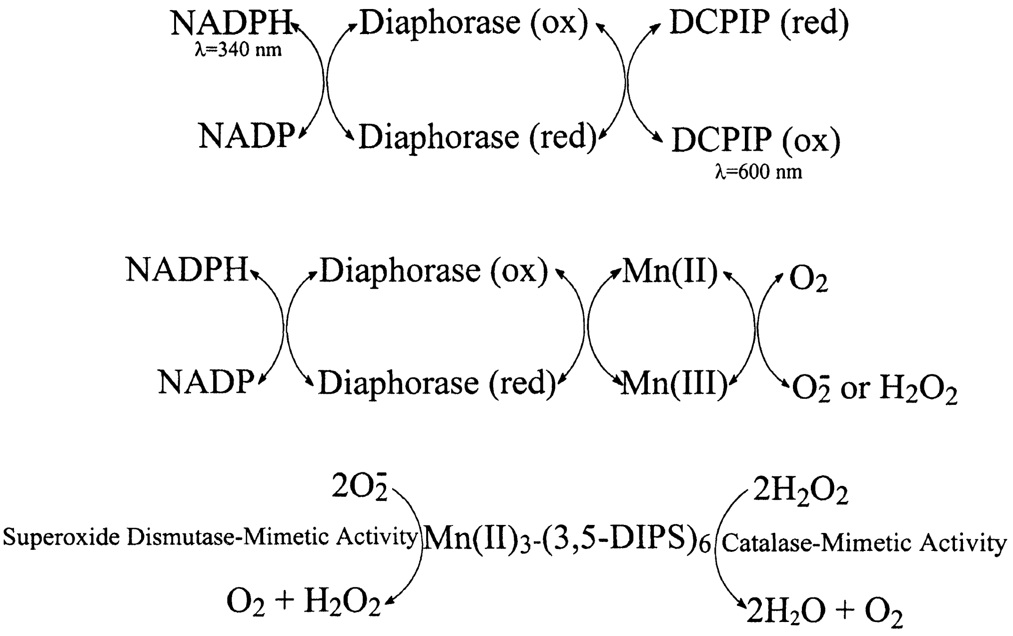

Figure 5. Schemes for the oxidation of reduced PHD in the presence of DCPIP and with its replacement with $Mn_3(3,5\text{-DIPS})_6$.

The net consumption of oxygen in these reactions could also offer an additional accounting for down-regulation of NOS which requires oxygen for the oxidation of L-Arg to L -Cit and NO. Disproportionation of superoxide by $Mn_3(3,5\text{-DIPS})_6$ may also appear to reduce superoxide produced by NOS when NOS functions to generate superoxide via a one electron reduction of molecular oxygen [21].

Recovery studies also demonstrated that large concentrations of NADPH impede the recovery activity of $Mn_3(3,5\text{-DIPS})_6$ which was not measurable in the system containing 114 μM NADPH with up to 48.8 μM $Mn_3(3,5\text{-DIPS})_6$. Lowering the concentration of NADPH to 38 μM allowed the measurement of PHD recovery. The presence of 114μM DCPIP may facilitate down-regulation of PHD by $Mn_3(3,5\text{-DIPS})_6$ by forming a DCPIP-Mn-3,5-DIPS complex shown in Figure 6. The down-regulation concentration for 50 percent decrease in DCPIP reduction ($DRC_{50}$) in the 114 μM NADPH and DCPIP system was 5 μM $Mn_3(3,5\text{-DIPS})_6$. However, the presence of 114 μM NADPH may inhibit or decrease the effectiveness of $Mn_3(3,5\text{-DIPS})_6$ in facilitation recovery of NADPH as a result of the formation of an NADPH complex (Figure 6).

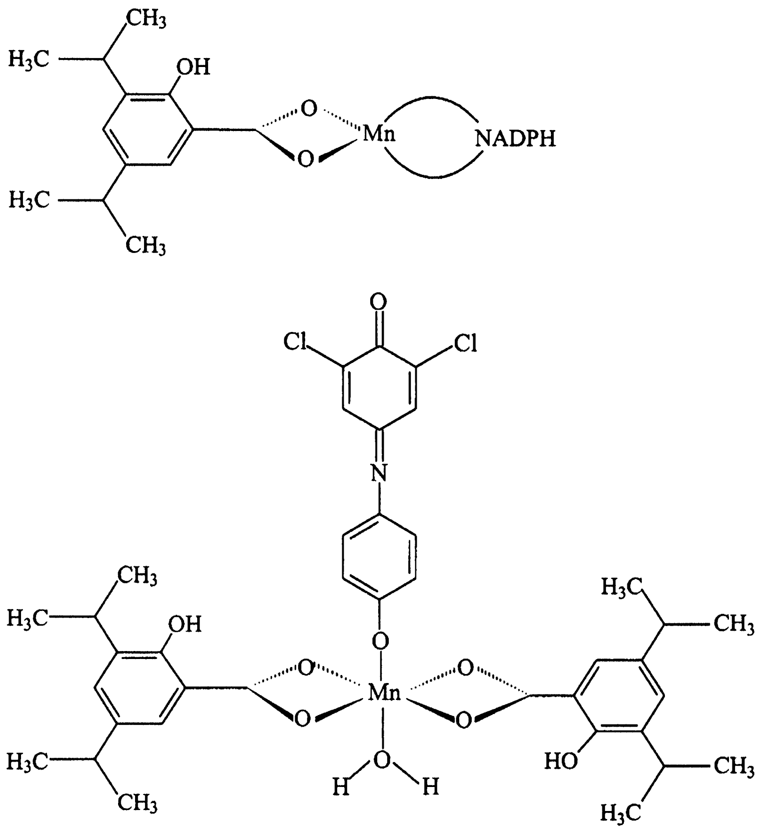

Figure 6. Proposed structure of the $Mn_3(3,5\text{-}DIPS)_6$-NADPH complex (top) that impedes PHD recovery activity when a large concentration of NADPH is present and the DCPIP-$Mn_3(3,5\text{-}DIPS)_6$ complex (bottom) that facilitates down-regulation of PHD when a large concentration of DCPIP is present.

Non-toxic doses of manganese complexes, which would supply less than the daily required intake of manganese, have been shown to have beneficial anticonvulsant effects in models of seizure and radiation injury [11,12]. Very small doses of $Mn_3(3,5\text{-}DIPS)_6$, have both radioprotectant and radiorecovery activities [12]. The plausible down-regulation of NOS by $Mn_3(3,5\text{-}DIPS)_6$ may account for its anticonvulsant activity in the Metrazole and Electroshock models of seizure [12]. In addition to Metrazole-induced [22] and electrically kindled seizures [23], seizures induced by N-methyl-D-aspartate (NMDA) receptor activation [24-26], tacrine [27], and sodium nitroprusside [$Na_2Fe(III)(CN)_5(NO)$] [26] are mediated via the NO-GC-NMDA pathway. However, pilocarpine-induced seizures were paradoxically facilitated by blockade of the NMDA receptor and inhibition of NOS [28]. Except for these as yet unexplained paradoxical results, all other results support hypotheses that NO synthesized by NOS from L-Arg and subsequent NO activation of GC leading to NMDA-receptor activation contribute to seizure states and that anticonvulsant activities of both $Mn_3(3,5\text{-}DIPS)_6$ are consistent with its possible down-regulation of NO synthesis.

Impedence of some step in the NOS-NO-cGMP-glutamate-NMDA-receptor pathway accounts for the anesthetic activity of ketamine, kainate, quisqualate, L-Glu, L-Asp [29], halothane [30-32], L-NAME [30], nitrous oxide, and isoflurane [32]. Decreased consciousness and sedation are also caused by interruption of this pathway [30] and may account for the anticonvulsant activity of $Mn_3(3,5\text{-}DIPS)_6$ and $Cu(II)_2(3,5\text{-}DIPS)_4$ [11].

Radiation injury is either a local or systemic inflammatory disease depending upon either focal-tissue or whole-body radiation exposure. Since NO has roles in perception of inflammatory insults and mediation of the host response to inflammation [10 and citations therein] and the modulation of NO synthesis is required to bring about cessation of this component of the inflammatory response, the down-regulation of NOS by $Mn_3$(3,5-DIPS)$_6$ and Cu(II)$_2$(3,5-DIPS)$_4$ may account for the increase in survival of lethally irradiated mice treated with these complexes [12,13].

It is generally accepted that NOS has features similar to Cytochrome p-450 in incorporating one atom of activated dioxygen into substrate and requiring activation of two molecules of dioxygen in the syntheses of L-Cit and NO from L-Arg [33-35]. However, this mechanism has been questioned by Leone et al. [36] who provided evidence consistent with NOS being a dioxygenase wherein both atoms of dioxygen are incorporated into L-Arg to give the two NOS products, NO and L-Cit. While both enzymatic transformations are plausible, the efficiency of a dioxygenase makes this possibility attractive. The isolation of $N^G$-hydroxy-L-arginine from a NOS reaction mixture may be a P-450-like oxidation product but a kinetically less likely product. In this event, $Mn_3$(3,5-DIPS)$_6$ being an effective SOD-mimetic or serving as an electron acceptor might also down-regulate this P-450 activity as has been shown for Cu(II)$_2$(3,5-DIPS)$_4$ and other Cu complexes [37-39].

Nitric oxide is a reactive molecule with a half-life of seconds in biological systems [40] and would most likely react with many cellular and extracellular components, causing marked chemical injury before it diffuses to a target cell. Since NO is reactive, NOS may include a component which accepts newly synthesized NO and serves as its transporter. Consequently, suggestions that NO is transported via either an organic thionitrite, nitrosoamine, alkyl and aryl nitrite, or peroxylnitrite or Fe, Mn, or Cu nitrosyl complexes [40,41] merit consideration and further study. While all of these potential transport forms of NO are plausible, evidence does exist for thionitrites of cysteine and penicillamine [41] in serving as organic NO transporting compounds and a NO complex of Fe, $Na_2Fe(III)(CN)_5(NO)$, is a well known NO transporting complex used to rescue patients in hypertensive crises. It is also plausible that nanomolar or lower concentrations of a Mn(II) or Cu(II) complex, smaller than the concentration that might down-regulate NOS, can serve as NO transporting complexes, O=N-Mn(III)$L_3$ or O=N-Cu(III)$L_3$ This point may be particularly relevant to the observation that the IC $_{50}$ far $Mn_3$(3,5-DIPS)$_6$ in decreasing NOS activity was 0.1 mM supporting the notion that a lower and more physiologically relevant concentration would be more likely to form a NO complex without decreasing NO synthesis. The reported [42] potentiation of the NO-mediated increase in cGMP by a small molecular mass Mn(II) complex (SC52608), while suggested to be due to its SOD-mimetic activity [42], could also be due to its ability to form a O=N-Mn(III) complex and serve as a NO transporting agent. There are many Mn-and Cu-dependent enzymes and their facitated activation by treatment with a Mn and or Cu complex also offers a plausible accounting for the pharmacological effects of low molecular mass Mn and Cu complexes [13]. Manganese- and Cu-dependent enzymes may also have a biochemical role in mediating normal physiologic roles of NO. Manganese and Cu requirements for normal physiological and biochemical function of NO-mediated events merit further investigation.

Acknowledgments

We are indebted to Harry P. Ward, Chancellor of the University of Arkansas for Medical Sciences Campus, the Winthrop Rockefeller Foundation, the Arkansas Medical, Dental, and Pharmaceutical Association, the National Institute of Health Research Apprenticeship Program, grant number 5R25RR10281-03 and a grant P11478 from the Fonds zur Foerdurung der Wissenschaftlichen Forschung in Austria for financial support.

References

[1] H. H. H. W. Schmidt, P. Wilke, B. Evers, E. Bohme, *Biochem. Biophys. Res. Comm.*, **165** (1989) 284.
[2] S. Moncada, R. M. J. Palmer, E. A. Higgs, *Pharmacol. Rev.*, **43** (1991) 109.
[3] S. Moncada, R.M.J. Palmer, *Trends Pharmacol. Sci.*, **12** (1990) 130.
[4] J. R. Lancaster, Jr., *Amer. Scient.*, **80** (1992) 248.
[5] S. T. Meller, G. F. Gebhart, *Pain*, **52** (1993) 127.
[6] A. K. Nussler, T. R. Billar, *J. Leuk. Biol.*, **54** (1993) 171.
[7] N. Miki, Y. Kawabe, K. Kuriyama, *Biochem. Biophys. Res. Comm.*, **75** (1977) 851.
[8] R. G. Knowles, M. Palacios, R. M. J. Palmer, S. Moncada, *Proc. Natl. Acad. Sci.USA*, **86** (1989) 5159.
[9] E. Southam, J. Garthwaite, *Neuropharmacol.*, **32** (1993) 1267.
[10] J. G. L. Baquial, J. R. J. Sorenson, *J. Inorg. Biochem.*, **60** (1995) 133.
[11] J. R. J. Sorenson, *Prog. Med. Chem.*, **26** (1989) 437.

[12] J. R. J. Sorenson, L. S. F. Soderberg, L.W. Chang, W. M. Willingham, M. L. Baker, J. B. Barnett, H. Salari, K. Bond, *Eur. J. Med. Chem.*, **28** (1993) 221.
[13] J. R. J. Sorenson, L. S. F. Soderberg, L.W. Chang, *Proc. Soc. Exptl. Biol. Med.*, **210** (1995) 191.
[14] B. O. West, *Polyhedron,* **8** (1978) 219.
[15] J. B. Vincent, H. R. Chang, K. Folting, J. C. Huffman, G. Christou, D. N. Hendericskon, *J. Am. Chem. Soc.*, **109** (1987) 5703.
[16] J. B. Vincent, H.-L. Tsai, A. G. Blackman, S. Wang, P. D. W. Boyd, K. Folting, J. C. Huffman, E. B. Lobkovsky, D. N. Hendrikson, G. Christu, *J. Am. Chem. Soc.*, **115** (1993) 12353.
[17] C. H. Harteneck, P. Klatt, K. Schmidt, B. Mayer, *Biochem. J.,* **304** (1994) 683.
[18] B. Mayer, P. Klatt, E. R. Werner, K. Schmidt, *Neuropharmacol.*, **33** (1994) 1253.
[19] P. Klatt, B. Heinzel, M. John, M. Kastner, E. Bohme, B. Mayer, *J. Biol. Chem.*, **267** (1992) 11374.
[20] P. A. Gonzalez, J. Zhuang, S. R. Doctrow, B. Malfroy, P. F. Benson, M. J. Menconi, M. P. Fink, *J. Pharmacol. Exptl. Therap.*, **275** (1995) 798.
[21] S. Pou, W. S. Pou, D. S. Bredt, S. H. Synder, G. M. Rosen, *J. Biol. Chem.*, **267** (1992) 24173.
[22] J. A. Ferrendelli, A. C. Blank, R. A. Gross, *Brain Res.,* **200** (1980) 93.
[23] M. E. Gilbert, *Brain Res.*, **463** (1988) 90.
[24] S. H. Synder, D. S. Bredt, *Trends Pharmacol. Sci.*, **12** (1991) 125.
[25] E. H. F. Wong, J. A. Kemp, T. Priestly, A. R. Knight, G. N. Woodruff, L. L. Iversen, *Proc. Natl. Acad. Sci. USA,* **83** (1986) 7104.
[26] G. De Sarro, E. D. Di Paola, A. De Sarro, M. J. Vidal, *Eur. J. Pharmacol.*, **230** (1993) 151.
[27] G. Bagetta, M. Iamnone, A. M. Scorsa, G. Nistico, *Eur. J. Pharmacol.,* **213** (1992) 301.
[28] M. S. Starr, B. S. Starr, *Pharmacol. Biochem. Behav.*, **45** (1993) 321.
[29] T. Yamamura, K. Harada, A. Okamura, O. Kemmotsu, *Anesth.*, **72** (1990) 704.
[30] R. A. Johns, J. C. Mosciki, C. A. DiFazio, *Anesth.*, **77** (1992) 779.
[31] Y-X Wany, A. Abdelrahman, C. C. Y. Pang, *J. Cardiovas. Pharmacol.*, **22** (1993) 571.
[32] R. W. McPherson, J. R. Kirsh, L. E. Moore, R. J. Traystman, *Anesth. Anal.,* **77** (1993) 891.
[33] D. J. Stuehr, N. S. Kwon, C. F. Nathan, O. W. Griffin, P. L. Feldman, J. Wiseman, *J. Biol. Chem.*, **266** (1991) 6259.
[34] K. A. White, M. A. Marletta, *Biochem.*, **31** (1992) 6627.
[35] P. Klatt, K. Schmidt, G. Uray, B. Mayer, *J. Biol. Chem.*,**268** (1993) 14781.
[36] A. M. Leone, R. M. J. Palmer, R. G. Knowles, P. L. Francis, D. S. Ashton, S. Moncada, *J. Biol. Chem.*, **226** (1991) 23790.
[37] C. Richter, A. Azzi, U. Weser, A. Wendel, *J. Biol. Chem.*, **252** (1977) 5061.
[38] U. Weser, C. Richter, A. Wendel, M. Younes, *Bioinorg. Chem.*, **8** (1978) 201.
[39] J. Werringloer, S. Kawano, N. Chacos, R. W. Eastabrook *J. Biol. Chem.*, **254** (1979) 11839.
[40] J. S. Stamler, D. J. Singel, J. Loscalzo, *Science,* **258** (1992) 1898.
[41] W. I. Rosenblum, *Stroke,* **23** (1992) 1527.
[42] T. P. Kasten, S. L. Settle, T. P. Misko, D. P. Riley, R. H. Weiss, M. G. Currie, G. A. Nickols, *Proc. Soc. Exptl. Biol. Med.*, **208** (1995) 170.

**Received: January 27, 1999 - Accepted: February 11, 1999 -**
**Received in revised camera-ready format: March 19, 1999**

